# Daytime versus Nighttime in Acute Appendicitis

**DOI:** 10.3390/diagnostics12040788

**Published:** 2022-03-23

**Authors:** Wouter J. Bom, Joske de Jonge, Jochem C. G. Scheijmans, Anna A. W. van Geloven, Sarah L. Gans, Marja A. Boermeester, Willem A. Bemelman, Charles C. van Rossem, on behalf of the SNAPSHOT

**Affiliations:** 1 Department of Surgery, Amsterdam UMC, Location AMC, Amsterdam Gastroenterology & Metabolism, University of Amsterdam, 1105 AZ Amsterdam, The Netherlands; j.c.scheijmans@amsterdamumc.nl (J.C.G.S.); s.l.gans@amsterdamumc.nl (S.L.G.); m.a.boermeester@amsterdamumc.nl (M.A.B.); w.a.bemelman@amsterdamumc.nl (W.A.B.); 2 Department of Surgery, TergooiMC, 1213 XZ Hilversum, The Netherlands; joskedejonge@gmail.com (J.d.J.); avangeloven@tergooi.nl (A.A.W.v.G.); 3 Department of Surgery, Maasstad Ziekenhuis, 3079 DZ Rotterdam, The Netherlands; rossemc@maasstadziekenhuis.nl

**Keywords:** complicated appendicitis, uncomplicated appendicitis, day, night

## Abstract

Background: Little is known about patients with appendicitis presenting at nighttime. It is hypothesized that patients presented at night more frequently have a complicated (gangrenous or perforated) appendicitis and therefore develop more postoperative complications. Methods: In this study data were used from the nationwide, prospective SNAPSHOT study appendicitis, including 1975 patients undergoing surgery for suspected appendicitis. This study included only adults. Two primary outcomes were defined: (A) The proportion of patients with complicated appendicitis and (B) the proportion of patients with a complication postoperatively presenting during daytime versus nighttime period. Analysis for both complicated and uncomplicated appendicitis was performed, and a multivariate model was used to correct for baseline characteristics and time to surgery. Results: In total, 1361 adult patients with appendicitis were analyzed. Both at nighttime and at daytime, 34% had complicated appendicitis. In patients presenting in the daytime, 12.1% developed a postoperative complication versus 18.6% for presentation at night (*p* = 0.008). In a multivariate analysis, the risk for a postoperative complication when presenting at night was significantly increased (adjusted OR 1.74; 95% CI 1.14–2.66, *p* = 0.01). Surgery within eight hours after presentation does not lower this risk (adjusted OR 1.37; 95% CI 0.97–1.95, *p* = 0.078). Conclusion: Complicated appendicitis is seen as frequently during the day as at nighttime. For patients who present at nighttime with acute appendicitis, the risk of a postoperative complication is higher compared with a presentation at daytime. In multivariate analysis, nighttime presentation but not surgery within 8 h after presentation is independently associated with postoperative complication risk.

## 1. Introduction

Appendicitis is a common cause of abdominal infections and has been extensively studied. Currently, it is thought that there are two types of appendicitis: complicated (gangrenous or perforated appendicitis) and uncomplicated (phlegmonous) appendicitis. Nevertheless, there is still controversy about the timing of appendectomy. A recent meta-analysis has demonstrated that delaying appendectomy for presumed uncomplicated appendicitis is not a risk factor of developing complicated appendicitis [1]. Moreover, it is safe to delay surgery for uncomplicated appendicitis up to 24 h without an increase of postoperative complications. These results suggest that surgeons can postpone appendectomy overnight in patients with suspected uncomplicated appendicitis [1]. On the other hand, guidelines advise that patients presenting with complicated appendicitis should be operated on within 8 h after diagnosis [2,3]. Interestingly, a study analyzing the prevalence of perforated appendicitis related to the time of presentation demonstrated that patients presenting between 09:00 and 15:00 have an increased risk of having perforated appendicitis up to 30% compared to early morning/late night presenters [4]. In the Netherlands, 50% of patients present during the day (between 08:00 and 18:00) and the other half presents in the evening or at night [5]. Therefore, it is essential to know if patients who present at night are the same type as patients presenting during the daytime, as patients who present later in the evening and at night are more likely to have their surgery postponed to the next day. 

Until now, no studies have described the characteristics and postoperative outcome of patients with acute appendicitis who present during daytime as compared to those at nighttime. Studies reporting on delayed appendectomy, only correct for time to surgery, unrelated to time of day [1]. The burden or threshold for a patient to go to the hospital is higher at night as compared to during the day. Therefore, patients with appendicitis who present at night may be more severely sick and thereby more likely to have a complicated disease type. In that case, delay of surgery may be less preferred during nighttime, at the very time that resources are diminished.

The aim of this study is to assess differences in characteristics and postoperative outcomes between patients with appendicitis who present during daytime versus those who present late in the evening or at nighttime. It is hypothesized that at nighttime, the proportion of patients with complicated appendicitis is higher and therefore have a higher risk of postoperative complications. Prospectively collected data from the Dutch SNAPSHOT appendicitis will be used to evaluate this hypothesis [5].

## 2. Materials and Methods

### 2.1. Study Design

Data was used from the SNAPSHOT appendicitis study, of which the full protocol has been published before [5]. This snapshot study is a prospective, observational, nationwide audit performed in The Netherlands in 62 Dutch hospitals, which included 1975 consecutive patients undergoing surgery for suspected appendicitis during a 2-month study interval (3 months in eight pilot centers; 219 inclusions in May, 887 in June and 869 in July 2014). The medical ethics committee of the Academic Medical Centre in Amsterdam approved the study design and judged that informed consent from patients was not necessary.

For the current study, a retrospective analysis was performed of the previously conducted prospective study. Only patients 18 years and older were included. Patients found to have a diagnosis other than appendicitis during surgery or who underwent surgery other than appendectomy were excluded. In addition, patients with unknown times of presentation at the emergency department were excluded.

### 2.2. Baseline Characteristics

The following baseline characteristics were reported: age, sex, days of complaints, presence of vomiting, signs of peritonitis during physical examination, ASA-classification, body temperature, C reactive protein (CRP), Leucocyte counts, suspicion of complicated appendicitis on imaging, time of presentation at the ED, and the time in hours between presentation at the ED and surgery. It was also reported whether surgery was performed by a resident without supervision, or by (or under supervision of) a consultant surgeon, surgical approach (laparoscopic or open) and what the operating time was.

### 2.3. Primary Outcomes

Two primary outcomes were defined:

- The proportion of patients with complicated appendicitis presenting during daytime period versus nighttime period;
- The proportion of patients with a postoperative complication for patients with acute appendicitis who present during daytime versus those at nighttime.

### 2.4. Secondary Outcomes

Baseline characteristics for complicated and uncomplicated appendicitis were reported. Other secondary outcomes were the following variables: proportion of patients with perforated appendicitis, a wound infection, another infectious complication, a re-intervention, a re-hospitalization, number of hospitalization days, time to surgery (hours), and mortality. These were compared for all patients with acute appendicitis who present at nighttime vs. those during daytime. This was analyzed separately for the subgroups of patients with complicated and uncomplicated appendicitis. The proportion of patients with any complications was also analysed separately for patients with complicated and uncomplicated appendicitis.

A comparison of postoperative complications was made in daytime versus nighttime among the strata ‘surgery performed within 8 h after presentation’ versus ‘surgery performed more than 8 h after presentation’. This was also performed for subgroups of patients with complicated or uncomplicated appendicitis. 

### 2.5. Definitions and Outcome Characteristics

Nighttime was defined as the time between 22:00 and 07:00 the subsequent morning; this definition of nighttime was used for time of presentation at ED and time of surgery. In contrast, ‘daytime’ was defined as the time between 07:00 and 22:00. Complicated appendicitis was defined as having perforated or gangrenous appendicitis, as described in the surgical report. Immediate postoperative start of antibiotic therapy was also viewed as indicative of a complicated form of appendicitis and therefore ranked as complicated appendicitis. This is according to the Dutch national guideline ‘Acute Appendicitis’ [3]. Complications were described as any adverse events during 30-days after surgery. Surgical site infections were classified as wound infections or abscesses. Re-interventions were defined as any surgical and/or radiological intervention. Time to surgery was the time between arrival time at the emergency department and the start of surgery. 

### 2.6. Data Analysis

Parametrical data were expressed in means with standard deviations, and unpaired T-tests were used to determine whether differences were significant. Non-parametrical data were expressed in medians with interquartile ranges, and a Mann–Whitney U test was used to determine significance. Chi-square was used for comparison of proportions, and if the expected count for one of the cells was below five, Fisher’s exact test was used. To analyze the proportion of complicated appendicitis per hour, chi-square was used. Significance was defined with a *p*-value of <0.05. A multivariate analysis was performed to determine whether the potential difference between patients who present at day and night remains significantly different or not. Variables added to the model were variables that predict complicated appendicitis (age, days of complaints, temperature, CRP, leucocyte count, and suspected complicated appendicitis on imaging) and baseline characteristics which might be considered as confounders. 

After selection variables with a *p* < 0.2, a multivariate binary logistic regression was performed. With a backward selection, variables with the highest *p*-value were removed from the regression until all variables had a *p* < 0.05. As patients presenting at nighttime as well as patients receiving surgery < 8 h were in our interest, these variables were fixed and would not be removed from the analyses. If data for multiple variables in the final model were missed in 10% of cases, data were imputed for the binary logistic regression.

## 3. Results

### 3.1. Baseline Characteristics

**Figure 1:**
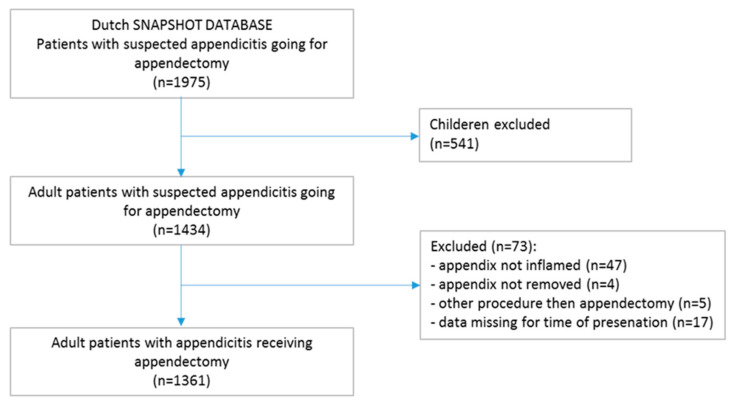
Flowchart of included patients.

From the database comprising 1975 patients with the clinical suspicion of acute appendicitis and with the intention of appendectomy, 541 children were excluded (Flowchart, Figure 1). Of 1434 adult patients, 1378 indeed received an appendectomy for appendicitis. From 56 patients who were operated but did not receive an appendectomy, the appendix was not inflamed in 47 patients. In the remaining nine patients, the appendix was not removed in four patients, because of a periappendicular inflammatory mass; in five patients, an ileocecal resection or right hemicolectomy was performed. In 17 patients, no time of presentation at the ED was administered. Therefore, 1361 patients were included in the present study.

**Figure 2:**
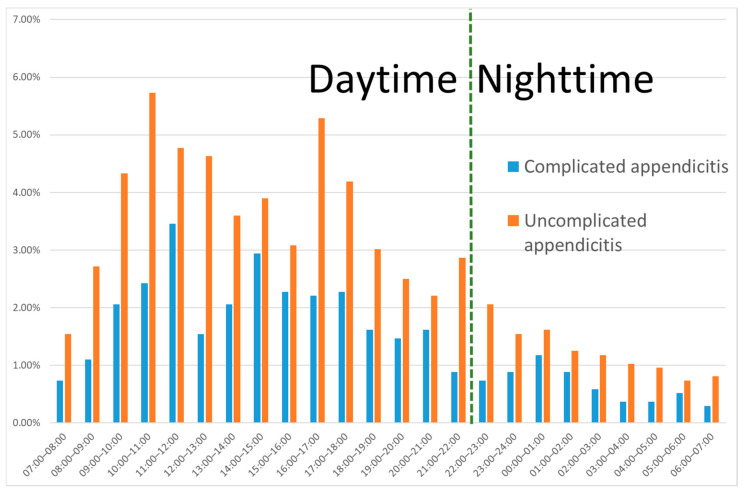
Hourly proportion (from total over 24 h) of patients with appendicitis presenting at the emergency department, for uncomplicated and complicated appendicitis.

Patterns for the time of presentation were comparable for complicated and uncomplicated appendicitis (Figure 2). Of 1361 patients with appendicitis who underwent appendectomy, 469 (34.5%) had complicated appendicitis and 892 (65.5%) uncomplicated appendicitis. Two hundred thirty-one patients (17%) presented at nighttime, 1130 (83%) at daytime. Patients who presented at nighttime were younger (33 years vs. 40 years, *p* < 0.001), more often experienced vomiting (43% vs. 33%, *p* = 0.005), had higher leukocyte counts (14.6 vs. 13.6, *p* = 0.004), lower CRP levels (20 vs. 41 mmol/L, *p* < 0.001) compared to patients who presented at daytime. Importantly, the nighttime patients were operated on less often within 8 h compared to daytime patients (15% vs. 64%, *p* < 0.001). Similar trends were seen for uncomplicated and complicated appendicitis (Table 1). Patients who presented at night were not operated on within eight hours more often when they had complicated appendicitis compared with patients with uncomplicated appendicitis (17.7% vs. 13.2%, *p* = 0.35).

### 3.2. Primary Outcome

**Figure 3:**
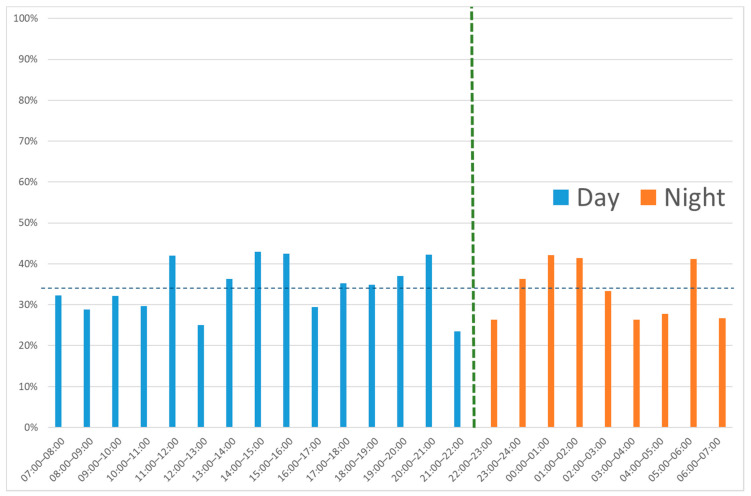
Proportion of patients with a complicated appendicitis relative to all patients with appendicitis per hour. Average difference day vs. night is 34.5% vs. 34.2% (*p* = 0.93), per hour *p* = 0.44.

1. At daytime, 390 of 1130 patients (34.5%) had a complicated appendicitis compared with 79 of 231 (34.2%) at nighttime (*p* = 0.93). No statistically significant difference in the proportion of patients with complicated appendicitis per daily hour was found (*p* = 0.44). (Figure 3).
2. Forty-three of 231 (18.6%) nighttime patients developed a postoperative complication versus 137 of 1130 (12.1%) daytime patients (*p* = 0.008) (Table 2).

### 3.3. All Appendicitis

For both complicated and uncomplicated appendicitis, most complications were caused by wound infections (Supplementary Table S1). Significantly more re-interventions were seen in patients who presented at nighttime versus those during daytime (6.1% vs. 2.4%, *p* = 0.003). 

In daytime patients, surgery within eight hours after presentation at the ED led to fewer complications compared to surgery after eight hours (10.4% vs. 15.1%, *p* = 0.02). For nighttime patients, the trend was in the opposite direction, but no significant difference was found comparing surgery within 8 h versus after 8 h (23.5% vs. 17.8%, *p* = 0.43). Postoperative complications were comparable for surgery during daytime versus surgery at nighttime (13.4% vs. 12.4%, respectively; *p* = 0.65). For patients operated by a consultant surgeon (or a resident under the direct supervision of one), 144 (13.1%) of 1099 patients developed a postoperative complication versus 36 (13.7%) of 262 patients, who were operated by a resident without supervising consultant (*p* = 0.78). Surgery by a resident without supervision did not increase the number of infections in the strata presentation at night versus day. 

### 3.4. Uncomplicated Appendicitis

For patients with uncomplicated appendicitis, 57 of 740 daytime patients (7.7%) developed a complication versus 18 of 152 (11.8%) nighttime patients (*p* = 0.09). Surgery within 8 h led to comparable results compared to surgery after 8 h in daytime as well as nighttime (7.1% vs. 8.7%, *p* = 0.42; 15.0% vs. 11.4%, *p* = 0.71, respectively).

### 3.5. Complicated Appendicitis

For patients with complicated appendicitis, significantly fewer daytime patients (80 of 290, 20.5%) developed a postoperative complication compared to nighttime patients (25 of 79, 31.6%; *p* = 0.03). During the daytime, surgery within 8 h led to significantly fewer complications compared to surgery after 8 h (17.3% vs. 25.8%, *p* = 0.043). At nighttime, there was no significant difference (35.5% vs. 30.8%, *p* = 0.76). 

### 3.6. Multivariate Analysis

The results from the multivariate analysis for all patients are shown in Table 3. Overall, 9.6% of cases were missing for this analysis, and, therefore, non-imputed data was used. Variables in the model after multivariate analysis were age, CRP, complicated appendicitis on imaging, surgery more than 8 h after presentation, and presenting at nighttime. The adjusted odds ratio for a postoperative complication of patients presenting at nighttime was 1.74 (95% CI 1.14–2.66, *p* = 0.010). For undergoing surgery more than 8 h after presentation at the emergency department, an adjusted OR of 1.37 (95% CI 0.97–1.95, *p* = 0.078) was found. 

Similarly, multivariate analyses were performed in the subgroups of complicated appendicitis and uncomplicated appendicitis. For complicated appendicitis, the only significant variable remaining in the model was age per year (adjusted OR 1.02, 95% CI 1.01–1.04, *p* < 0.001). Surgery after 8 h and presentation at nighttime both were not significantly associated with postoperative complications (adjusted OR 1.47 (95% CI 0.92–2.36, *p* = 0.11) and 1.58(95% CI 0.89–2.80, *p* = 0.12), respectively). For uncomplicated appendicitis, age (adjusted OR 1.02, 95% CI 1.003–1.032, *p* = 0.02) and surgery by a resident without supervision (adjusted OR1.93, 95% CI 1.14–3.26) were the only significant variables associated with postoperative complication remaining in the model after multivariate analysis. Again, adjusted odds ratios for surgery after eight hours and for presentation at nighttime were not significantly associated (adjusted OR 1.12 (95% CI 0.66–1.93, *p* = 0.66) and 1.65 (95% CI 0.87–3.14, *p* = 0.13), respectively).

## 4. Discussion

Based on the SNAPSHOT appendicitis data, patients presenting at night were younger, vomited more often, had lower CRP levels, higher leucocytes and were often not operated on within eight hours after presentation. The percentage of adults with complicated appendicitis presenting at the ER during nighttime was comparable to that of patients who present in the daytime. Nevertheless, patients who presented at night developed a complication postoperatively more frequently than patients who presented during daytime (18.1% vs. 12.1%, *p* = 0.008). This difference in complication rate was predominantly observed for the complicated appendicitis subgroup but was not associated with a delay in surgery beyond 8 h after ED presentation. 

In a multivariable model adjusted for possible confounders, surgery within eight hours did not decrease the proportion of complications significantly. Patients who presented at nighttime still had a significantly higher chance of a complication than those who presented during daytime. 

A possible explanation may be that nighttime operations were performed predominantly in the more severely ill patients presenting at nighttime, having an inherently higher risk of a postoperative complication. However, this was not confirmed in our data. Another explanation is that patients who present at night are diagnosed later, as their imaging may be delayed until early morning. Moreover, these patients probably did not get any antibiotics while waiting for diagnosis until subsequent surgery. This may have increased the chance of a complication. Unfortunately, our data lack sufficient detail to explore underlying explanatory mechanisms. 

As no other studies have correlated the clinical outcome after appendectomy with the time of presentation, our data could not be compared to other results. Drake et al. [4] described that patients who present midday had a higher risk of perforated appendicitis. Looking at their data shows that differences are minor, with percentages fluctuating between 11 and 25% per daily hour. They found a significant, minor difference in groups per 4 h, which seems to disappear if groups are compared per hour. By eyeballing, no clear pattern is seen in their data. Differences in postoperative complications are not reported in that study.

The strengths of the present study include the definition of complicated appendicitis. Using the intraoperative diagnosis as the reference standard for complicated appendicitis results are closer to an intention to treat analysis rather than defining the reference standard according to pathology results. Furthermore, intraoperative outcomes are a better predictor of postoperative complications [6]. Secondly, the prospective and nationwide character of collecting data makes sure that no patients were missed in the given time period. It represents the daily clinical practice in the Netherlands.

Possible limitations include the fact that children were excluded. This was done as the decision to go to the ER is influenced by parents and not only by the patient. A second limitation is that the data was collected by many residents, leading to possible inter-observer variability; this is due to the SNAPSHOT design. On the other hand, it represents the best data reporting about daily clinical practice. A third limitation is the missing data for possible antibiotic treatment given at the ED or ward before surgery. 

Complicated appendicitis is not seen more frequently at nighttime than during the day. Patients presenting with acute appendicitis at the emergency department at nighttime have more postoperative complications. Exploring various subgroups and variables, no clear explanation was found for this increased postoperative complication rate at nighttime presentation. Surprisingly, surgery within eight hours after presentation at nighttime was not independently associated with a lower risk of postoperative complications.

## Figures and Tables

**Table 1:** Baseline characteristics.

	% Missing	All Appendicitis (*n* = 1361)			Uncomplicated Appendicitis (*n* = 892)			Complicated Appendicitis (*n* = 469)		
		Day (*n* = 1130)	Night (*n* = 231)	*p*-Value	Day (*n* = 740)	Night (*n* = 152)	*p*-Value	Day (*n* = 390)	Night (*n* = 79)	*p*-Value
Age (years)	0%	40 (28–55)	33 (25–48)	<0.001	36 (25–50)	29 (22–42)	<0.001	50 (35–62)	49 (32–63)	0.68
% male	0%	50.2%	58.1%	0.55	49.7%	46.1%	0.41	51.0%	51.9%	0.89
Days of complaints	2.2%	1 (1–2)	1 (1–2)	<0.001	1 (1–2)	1 (1–1)	<0.001	2 (1–3)	1 (1–1)	0.15
Absence of diffuse peritonitis	3.7%	90.1%	91.0%	0.69	93.6%	95.9%	0.28	83.5%	81.3%	0.65
Migrating pain	2.9%	44.0%	40.4%	0.32	44.7%	45.0%	0.95	42.8%	31.6%	0.069
Vomiting	2.2%	33.1%	42.7%	0.005	27.6%	37.3%	0.017	43.5%	53.2%	0.12
ASA 1 or 2	0%	95.8%	98.3%	0.077	96.5%	100%	0.014 ^#^	94.6%	94.9%	1.0 ^#^
Temperature (°C) *	5.6%	37.4 (0.8)	37.4 (0.7)	0.57	37.3 (0.7)	37.4 (0.7)	0.034	37.7 (0.8)	37.5 (0.7)	0.108 *
Leucocytes (10^9^/L) *	0.1%	13.6 (4.5)	14.6 (4.5)	0.004	13.1 (4.4)	14.2 (4.2)	0.007	14.5 (4.4)	15.3 (5.0)	0.194 *
Complicated appendicitis on imaging	0%	15.9%	16.0%	0.97	6.6%	7.2%	0.78	33.6%	32.9%	0.91
CRP (mmol/L)	0.4%	41 (16–88)	20 (6–66)	<0.001	30 (11–60)	13 (4–38)	<0.001	86 (36–173)	68 (17–141)	0.049
Time to surgery (h)	0.6%	6.3 (4.3–10.8)	13.9 (10–17)	<0.001	6 (4–11)	14 (11–17)	<0.001	6 (4–11)	14 (10–18)	<0.001
Surgery < 8 h	0.6%	64.1%	14.7%	<0.001	66%	13.2%	<0.001	61.1%	17.7%	<0.001
Surgery by resident without supervision	0%	18.4%	23.4%	0.081	19.7%	28.3%	0.019	15.9%	13.9%	0.66
Surgical time (min)	4.5%	42 (32–55)	45 (35–57)	0.11	40(30–50)	42 (32–54)	0.06	48 (36–64)	51 (37–60)	0.91
Laparoscopic surgery	0%	79.2%	81.8%	0.37	79.1%	82.9%	0.28	79.5%	79.7%	0.96

Numbers are reported in median (interquartile range) or percentages * Normally distributed, reported as average (standard deviation), unpaired *t*-test was performed. ^#^ Expected count in one of the cells < 5, therefore Fisher’s exact test was performed.

**Table 2:** Postoperative outcomes.

	All Appendicitis (*n* = 1361)			Uncomplicated Appendicitis (*n* = 892)			Complicated Appendicitis (*n* = 469)		
	Day (*n* = 1130)	Night (*n* = 231)	*p*-Value	Day (*n* = 740)	Night (*n* = 152)	*p*-Value	Day (*n* = 390)	Night (*n* = 79)	*p*-Value
Perforated appendicitis ^1^	19.1%	22.1%	0.30	-	-	-	55.4%	64.6%	0.13
Complications (overall)	12.1%	18.6%	0.008	7.7%	11.8%	0.09	20.5%	31.6%	0.03
Infectious complications	6.8%	10.8%	0.04	4.2%	5.9%	0.35	11.8%	20.3%	0.04
Mortality	0.2%	0%	1.0 ^#^	0.1	0	1.0 ^#^	0.3%	0%	1.0 ^#^
Reinterventions	2.4%	6.1%	0.003	1.4%	3.9%	0.04 ^#^	4.4%	10.1%	0.05 ^#^
Hospitalization days	2 (1–4)	2 (1–4)	0.89	2 (1–2)	2 (1–2)	0.46	5 (3–6)	4 (3–7)	0.70
Re-admission	5.5%	8.7%	0.07	4.5%	7.9%	0.08	7.4%	10.1%	0.42

^1^ Diagnosed intraoperatively ^#^ Fisher exact test.

**Table 3:** Multivariate analysis for a postoperative complication.

	Univariable Odds Ratio			Multivariate Adjusted Odds Ratio		
	OR	95% CI	*p*-Value	OR	95% CI	*p*-Value
Age (per year)	1.03	1.02–1.04	<0.001	1.02	1.01–1.03	<0.001
Days of complaints	1.14	1.05–1.24	0.001	-	-	-
Diffuse peritonitis	1.71	1.07–2.73	0.025	-	-	-
Vomiting	1.08	0.77–1.50	0.66	-	-	-
ASA 3 or 4	2.89	1.55–5.40	0.001	-	-	-
Temperature (per °C)	1.31	1.06–1.60	0.01	-	-	-
Leucocytes (per 10^9^/L)	1.03	0.99–1.06	0.11	-	-	-
CRP (per mmol/L)	1.004	1.003–1.006	<0.001	1.003	1.001–1.005	0.002
Complicated appendicitis on imaging	2.26	1.56–3.25	<0.001	1.67	1.13–2.48	0.01
Surgery > 8 h	1.54	1.12–2.11	0.008	1.37	0.97–1.95	0.078
Presenting at nighttime	1.66	1.14–2.42	0.008	1.74	1.14–2.66	0.010
Surgery by resident without supervision	1.06	0.71–1.57	0.78	-	-	-

## Data Availability

The data presented in this study are available on request from the corresponding author. The data are not publicly available due to privacy reasons.

## Supplementary Materials

The following supporting information can be downloaded at: https://www.mdpi.com/article/10.3390/diagnostics12040788/s1, Table S1: All complications.
