# Supplementary material for: Daytime versus Nighttime in Acute Appendicitis
Source: Diagnostics (Basel). 2022 Mar 23;12(4):788. doi: 10.3390/diagnostics12040788 (PMC9028960; doi:10.3390/diagnostics12040788)
Supplement: Supplementary file 1 [file diagnostics-12-00788-s001.zip › diagnostics-1608391-supplementary.pdf]

Table S1. All complications.

	All Appendicitis (n = 1361)			Uncomplicated Appendicitis (n = 892)			Complicated Appendicitis (n = 469)		
	**Day (n = 1130)**	**Night (n = 231)**	***p*-value**	**Day (740)**	**Night (n = 152)**	***p*-value**	**Day (390)**	**Night (n = 79)**	***p*-value**
Complications (overall)	12.1%	18.6%	0.008	7.7%	11.8%	0.09	20.5%	31.6%	0.03
Infectious complications (grouped)	6.8%	10.8%	0.04	4.2%	5.9%	0.35	11.8%	20.3%	0.04
Woundinfection	3.5%	4.8%	0.34	2.7%	3.9%	0.43#	4.9%	6.3%	0.58#
Intra-abdominal abcess	3.6%	7.4%	0.01	1.8%	3.3%	0.21#	7.2%	15.2%	0.02
Ileus	1.7%	2.6%	0.42#	0.7%	0.7%	1.0#	3.6%	6.3%	0.34#
Pneumonia	0.4%	0.4%	1.0#	0.3%	0%	1.0#	0.5%	1.3%	0.43#
Urinary tract infection	0.4%	0%	1.0#	0.3%	0%	1.0#	0.5%	0%	1.0#
Bleeding complication	0.5%	0%	0.60#	0.3%	0%	1.0#	0.4%	0%	1.0#
Cardiopulmonal failure	0.7%	0%	0.37#	0%	0%	1.0#	2.1%	0%	0.36#
Death	0.2%	0%	1.0#	0.1%	0%	1.0#	0.3%	0%	1.0#
Other	0.4%	2.2%	0.02#	0.3%	1.3%	0.14#	0.8%	3.8%	0.06#

Numbers are reported in percentages; # Expected count in one of the cells <5, therefore fisherman exact test was performed
